# The ‘FRIENDS for Life’ emotional health programme: Differential impact for those at risk

**DOI:** 10.1111/bjep.70005

**Published:** 2025-06-27

**Authors:** Michael Wigelsworth, Margarita Panayiotou, Garry Squires, Karolina Byc

**Affiliations:** ^1^ Manchester Institute of Education The University of Manchester Manchester UK

**Keywords:** affective processes, cognitive processes, contexts of learning, primary/elementary schools, self‐regulation, stress and coping

## Abstract

**Background:**

Evidence suggests that FRIENDS, a universal cognitive behavioural programme for schools, can improve children's emotional health, yet debate persists regarding its efficacy with respect to prevention versus treatment, particularly for children at risk of anxiety disorders.

**Aim:**

To examine the impact of FRIENDS across different risk categories by assessing: (a) changes in risk status resulting from intervention and (b) treatment effects within specific risk groups.

**Sample and Methods:**

Secondary analysis of data from a cluster randomized trial (ISRCTN13721202) conducted between 2016 and 2018 involving approximately 3000 pupils (aged 9–10) from 79 schools. Self‐reported anxiety, depression and worry measures were collected at pre‐ and post‐test. Risk categories were established using baseline anxiety and depression scores.

**Results:**

FRIENDS did not significantly change risk status, neither reducing risk (*χ* ^2^ (1) = 1.667; *p* =.797) nor preventing progression to higher risk categories (*χ* ^2^ (1) = .44; *p* =.507). Within risk categories, significant effects appeared only in the clinical risk group (*β* = 1.83 (*SE* = .14), *d* = .67), with no significant effects for borderline (*β* = 1.03 (*SE* = .98), *d* = .18) or normal (*β* = .03 (*SE* = .33), *d* < .01) categories.

**Conclusion:**

While FRIENDS did not alter risk status, there appears treatment effects specifically for children within the clinical range for anxiety and depression. Findings provide a more nuanced understanding of who benefits from universal school‐based interventions. Findings inform health and education professionals in balancing FRIENDS’ treatment effects against factors like availability of alternative services, relative costs and sustainability.

## BACKGROUND

Anxiety and depression are among the most common mental health disorders experienced by children. Although depression and anxiety have unique symptoms, there is a high degree of comorbidity (Cummings et al., 2014), with symptomology characterized by low mood, loss of interest or pleasure, or notable sadness (depression), persistent feelings of unease or fear (anxiety) and/or poor concentration, impacting daily life (Brady & Kendall, 1992). Children's general anxiety symptoms can also manifest as excessive worry (with symptomology including feeling on edge, fatigue, difficulty concentrating, muscle tension, irritability or difficulty sleeping) that interferes significantly with psychosocial functioning (DSM‐5; American Psychiatric Association, 2013).

It is suggested that by the age of 16, up to 20% of children will have been diagnosed with an anxiety and/or depressive disorder (Solmi et al., 2022), with many more children experiencing serious symptoms that fall below clinical criteria for diagnosis. Sustained experience of anxiety and depression may negatively impact children's academic attainment (McCurdy et al., 2023) and as mental health disorders are also often seen to persist into adulthood (Mulraney et al., 2021), there is a risk of lifelong difficulties for children experiencing early difficulties without early intervention. However, most children and young people experiencing these difficulties do not have access to comparable levels of support (Crenna‐Jennings & Hutchinson, 2020), with prevalence as low as one in three in receiving treatment in some contexts (Baker & Kirk‐Wade, 2024). There are multiple reasons for this lack of uptake, including a shortage of available support services, leading to a ‘wait to fail’ model in which access is prioritized for those with significant, disruptive difficulties. This is particularly problematic in the context of anxiety and depression given the nature of internalizing disorders which are often undetected until the point difficulties become entrenched (Missenden & Campbell, 2019), impacting daily living. This means early intervention and prevention during early adolescence are critical to altering the trajectory of mental health difficulties (McGorry & Mei, 2018), especially in specific relation to anxiety and depression. As such, schools and classrooms have become a popular nexus for intervention, given (for example) the comparatively convenient and universal access to the population of interest (Hoover & Bostic, 2021). There is broad evidence supporting the ability of classroom‐based intervention and prevention programmes to reduce the severity or prevent the onset of anxiety and depressive symptoms early in the life course (Fisak et al., 2023) through the acquisition, reinforcement and maintenance of specific skills and behaviours designed to have preventative ‘downstream’ effect anxiety and depression symptoms.

**FIGURE 1:**
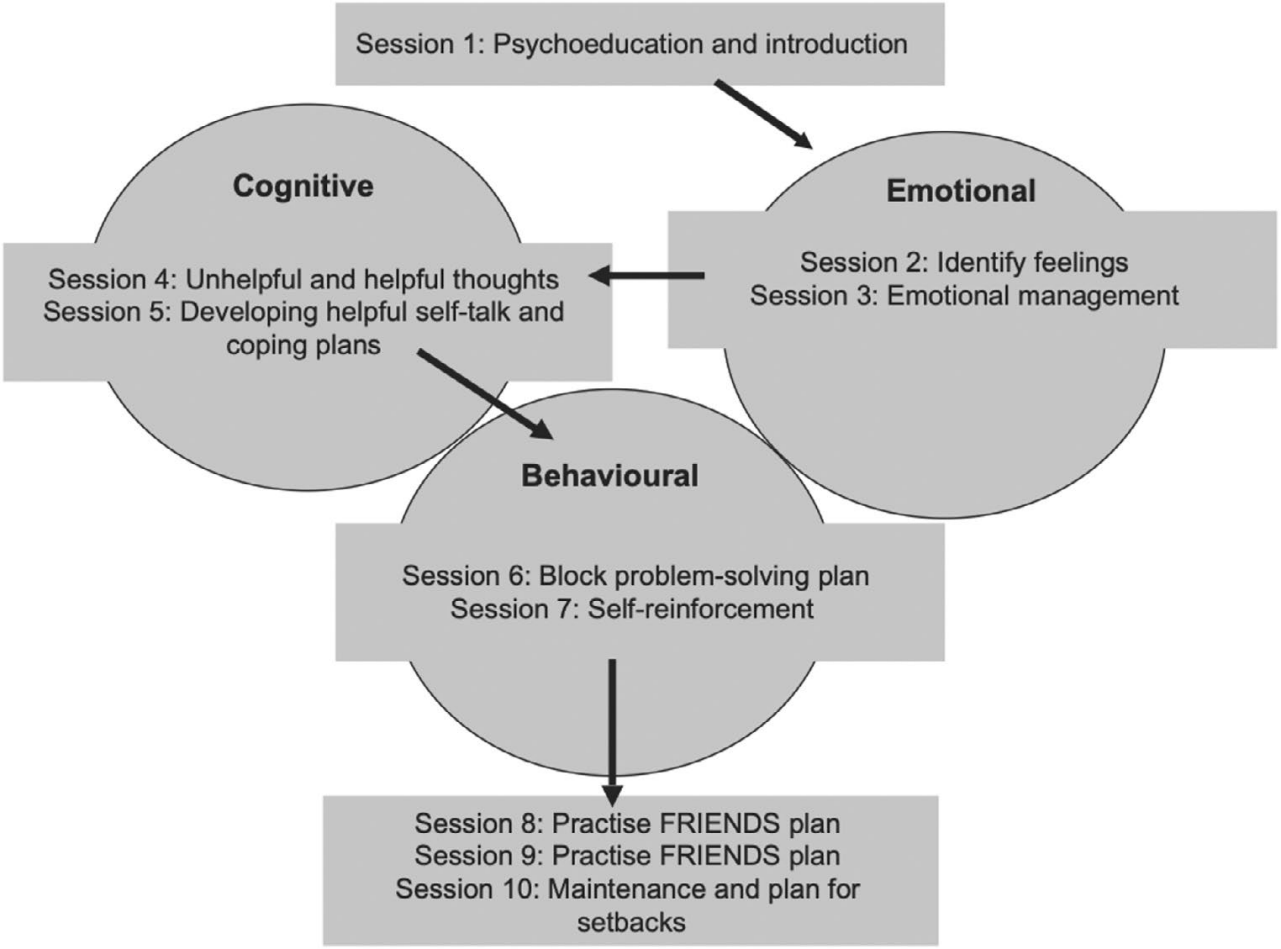
FRIENDS programme structure (adapted from Stallard, 2010).

In the context of early prevention of mental health difficulties in children and adolescents, cognitive behavioural therapy (CBT) is one of the evidenced approaches available (Halder & Mahato, 2019) and has been referred to as the ‘gold standard’ in psychological treatment (David et al., 2018). CBT is based on being able to identify and define problematic emotive states, thoughts and behaviours, and to elicit the appraisal of scenarios or circumstances that are associated with these difficulties. Individuals are supported to develop alternative theoretical accounts through a cognitive appraisal approach to allow individuals to develop strategies for addressing problematic thoughts, utilize alternative appraisals in given contexts and develop maintenance skills to reinforce and generalize strategies (Salkovskis et al., 2023). Based on a broad framework, the implementation of CBT comes in several diverse variants and contexts (e.g., hospital or community settings, individual or family formats), raising concerns about the generalizability of evidenced approaches. Of particular relevance to the context of education and that of early prevention is the core point that such approaches are often delivered and evaluated based on whole‐class delivery *regardless of need* (e.g., an explicit curriculum of content and instruction based on Figure 1 is delivered over a number of weeks as part of an explicit school lesson). Accordingly, there is currently very little understanding with respect to any differential impact for students who may already be experiencing mental health difficulties, students at risk or showing early symptomology or students who may be able to draw upon any current experiences of problematic states, learning CBT strategies to be deployed later in the life course. Given the potential heterogeneity of a given class receiving the whole‐class intervention, there is a question as to the associated extent to which such a delivery model may be effective for these groups, raising questions as to the cost–benefit of a whole‐class approach (e.g., how responsive are different groups to a class‐based intervention?).

### Early intervention and prevention

Early intervention and prevention approaches are typically classified as either universal, selective or indicated, differentiated on the basis of population needs (Foxcroft, 2014). Universal approaches are applied to all, regardless of need (e.g., whole class), whereas selective approaches are targeted at those at enhanced risk of developing problems (e.g., children already known to school) and indicated approaches are considered as early intervention for those already showing symptomology (e.g., identified through regular screening or by teacher referral). Distinctions between approaches typically include the specialist training of the staffing (with selective or indicated provision often having higher training requirements and/or delivered by external staff) and dosage (with selective or indicated provision often requiring more intensive commitments). Early intervention and prevention are akin to ‘inoculation’, that acquiring strength‐based skills early on in the developmental life course leads to significant benefits in relation to the costs (e.g., societal, economical and moral) of later treatment (e.g., cost of mental health treatment) as well as ensuring non‐stigmatizing coverage of otherwise sensitive topics.

Universal early intervention approaches present a number of dilemmas in tailoring optimal intervention. For instance, the theorized mechanisms of change relating to the acquisition and maintenance of relevant skills may be less effective for children not experiencing symptomology. Typical skill acquisition includes recognizing maladaptive conditions and avoiding behaviours to deploy strategies such as physiological relaxation and cognitive restructuring, which are difficult to effectively practice and maintain if the initial triggering behaviour (e.g., feeling anxious) is not a prevalent experience for the individual. Although there is strong evidence for the use of the approaches for those experiencing symptomology (Thielemann et al., 2022), there is far less evidence supporting instruction in reinforcement techniques as a preventative technique, presenting a potential ‘cart before horse’ scenario and indicating a potential opportunity cost (e.g., missed lesson time covering other policy‐relevant areas). This is especially true in the context of a relatively low prevalence of psychopathology among children, meaning many of those receiving universal intervention would not have otherwise developed later mental health concerns.

Conversely, in some instances (e.g., for the small number of children who may be experiencing emergent difficulties) universal provision can be criticized for being insufficient in regard to the dosage or intensity required to adequately address the developmental pathways of those affected (i.e., too ‘light touch’) (Maggin & Johnson, 2014). In this instance, although the mechanisms of change may be consistent, the extent to which opportunities for reinforcement or practice may be insufficient. For instance, research has shown a positive relationship between exposure exercises and symptom reduction (Whiteside et al., 2020). In these instances, effective resourcing would arguably be better deployed through selected or indicated approaches.

Accordingly, understanding how best to tailor the optimal level of support for recognized needs is critically important in a policy context whereby rationing of finite resources is necessary.

### The FRIENDS programme

Of particular interest in the examination of optimal levels of support is the FRIENDS programme (Appendix A), which reports to both prevent and address early symptomology of anxiety and depression (Barrett, 2005). ‘FRIENDS for life’ (hereafter referred to as FRIENDS) is a universal, psychoeducational programme based on CBT. Consistent with CBT theory, FRIENDS teaches the acquisition, reinforcement and maintenance of specific skills and behaviours designed to have preventative ‘downstream’ effects on anxiety, depression and worry symptoms and behaviours. Skills taught include behavioural responses to emotional (e.g., identify and manage anxiety), cognitive (e.g., identify and challenge anxiety‐increasing cognitions) and physiological (e.g., controlled breathing exercises) stimuli (see Figure 1). A breakdown of the sessional content with examples of how this relates to cognitive behavioural change can be seen in the supplementary material accompanying this paper.

FRIENDS is one of the most prevalent anxiety prevention programmes in the world for children and adolescents (Doyle et al., 2020; Maalouf et al., 2020; Najmussaqib et al., 2024) showcasing a significant international evidence base, with dozens of empirical studies showing a reduction in anxiety and/or depression across a number of different countries (see Fisak et al., 2023). Although reducing anxiety and depression are often the outcomes most closely associated with FRIENDS, in part due to their recognition as categorically and clinically relevant conditions across the life course (Morales‐Muñoz et al., 2023), worry is also considered relevant in the context of the FRIENDS intervention (Stallard et al., 2015). Defined as a predominance of verbal thought that leads to cognitive avoidance and inhibits emotional processing (Borkovec et al., 1998), recent research has positioned worry as a transdiagnostic factor for psychopathology within the areas of depression and anxiety (Wallsten et al., 2023). Such positioning is extremely relevant for early intervention as a potential precursor indicator of risk for developing anxiety and depression symptoms.

FRIENDS is currently endorsed by agencies such as the Substance Abuse and Mental Health Services Administration and the World Health Organization (2004). Although the weight of evidence implies FRIENDS can *potentially* be effective in reducing early symptoms of worry, anxiety and/or depression already present and/or lowering the prevalence of the emergence of symptomology later in the life course, there is an ambiguity as to who precisely benefits in respect to the aforementioned taxonomy of intervention (broadly, every pupil regardless of risk status (universal), those at risk (selective), or those with identifiable symptomology (indicated)), meaning the success of the FRIENDS intervention may be unfairly overgeneralized. For instance, although FRIENDS is described as a universal provision, some studies have exclusively focused on high‐risk pupils who demonstrated elevated difficulties at baseline (Bernstein et al., 2005; Miller et al., 2011; Pereira et al., 2014; Siu, 2007; Wergeland et al., 2014).

Results from other studies show a positive impact for students not otherwise at high risk for developing mental health difficulties (Gallegos‐Guajardo et al., 2013). Although meta‐analytic evidence suggests a differential effect for those different cohorts, with an overall higher impact of FRIENDS for high‐risk students (Hedges *g* =.37) in comparison to low‐risk students (Hedges *g* = −.26) (Maggin & Johnson, 2014), meta‐analyses are vulnerable to issues of heterogeneity (Eysenck, 1994). Inarguably the most contemporary and robust meta‐examination of over 40 trials of FRIENDS, Fisak et al. (2023) fail to consider the heterogeneity of samples within universal delivery modes (i.e., reporting a single effect size for class‐based delivery of FRIENDS despite the high likelihood of differential rates of impact as theorized above).

With a general failure of current literature to identify and examine the impact of FRIENDS on different risk groups and difficulties in reconciling different thresholds for risk status across studies despite its saliency, there remains a question as to the differential impact of FRIENDS on different risk groups more explicitly.

As the weight of evidence suggests increased intervention effects for those at risk, this study aims to examine the potential differential impact of the FRIENDS programme when delivered across a large number of English primary schools by educators. In doing so, the study also intends to add to the debate as to the suitability of educators in delivering FRIENDS (Briesch et al., 2010; Doyle et al., 2020; Fisak et al., 2023), as earlier studies have failed to find an effect on mental health outcomes when compared to health‐led outcomes (e.g., background in Psychology or other allied fields) (Skryabina et al., 2016). Although trials have taken place in this context (Stallard et al., 2014), limitations in sample size have prevented a closer examination of subgroup differences. In this study, we offer a significant increase in sample size in comparison to previous trials, being among the first to examine differential impact on high‐risk children when FRIENDS is delivered as a universal intervention by educators. In considering further potential differential impact of FRIENDS, we hope to better understand optimal treatment and prevention strategies with respect to universal provision and school‐based intervention by education staff.

### Aims

The aim of the study was to identify any differential impact of the intervention for different risk categories of children. This was approached in two main ways.

By considering whether there is a change in risk status as a result of FRIENDS (i.e., pupils moving *across* groups of risk as a result of intervention).
Whether there was any evidence of treatment effects *within* risk status (i.e., a difference in the magnitude of effect for post‐test scores for different risk groups).

## METHOD

### Trial design

**FIGURE 2:**
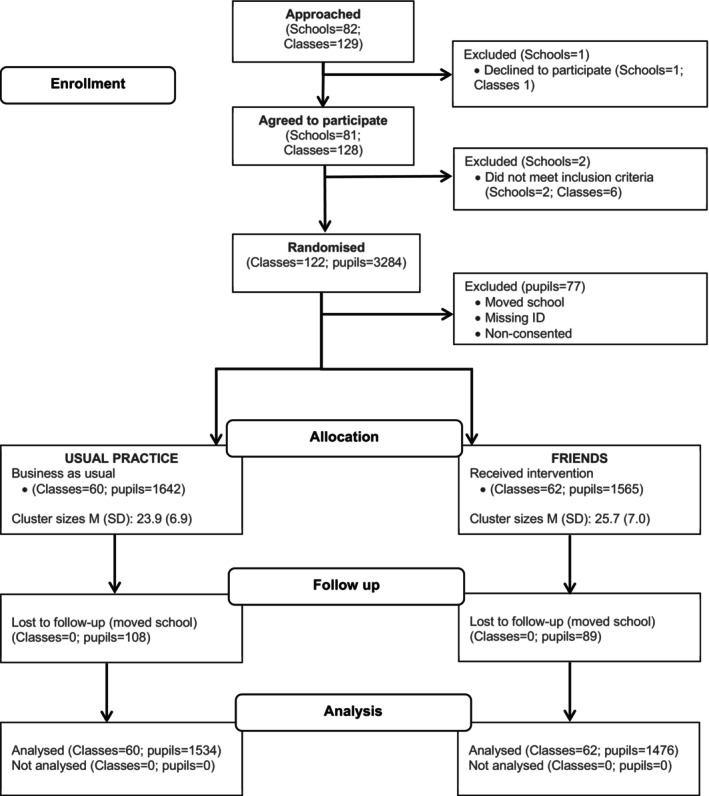
CONSORT flow diagram.

This study utilized secondary analysis of data from a cluster randomized control trial of FRIENDS, delivered in 79 English primary schools between April and July 2016 (ISRCTN13721202) (Figure 2). The original analyses tested the impact of FRIENDS on the attainment of pupils, measured by combined Key Stage 2 English and Maths scores. The project found no evidence that FRIENDS had a positive impact on children's academic attainment overall, though for pupils eligible for free school meals, those in the FRIENDS classes made 1 additional month's progress on a combined maths and reading measure compared to children in other classes (Wigelsworth, 2018). Ostensibly, key questions outside the scope of the originally funded research but fundamentally integral to understanding FRIENDS and its potential impact (i.e., differential impact for different risk categories of pupils when delivered as a universal intervention) remain unanswered. Additional analyses addressing these gaps in knowledge were conducted and reported in the current study.

The original study included a comprehensive implementation and process evaluation. Although examination of implementation data is beyond the scope of the current study (e.g., sample size limitations prevent including implementation measures and subgroup analysis simultaneously) the original study identified some evidence for a relationship between implementation processes and pupil outcomes, specifically an identified association between quality of delivery and reduction in pupils’ self‐rated worry (ES = −.469, CI = −.523 to −.415), while level of engagement paradoxically showed a smaller but significant effect in the opposite direction (ES = .237, CI = .185 to .289). Implications of implementation and process are considered further in the discussion.

For allocation to condition, block randomization was used (Festing, 2020) (using a number of classes within a year group – single; double; triple; quad) alongside minimization (Treasure & MacRae, 1998) (using prior attainment and baseline ‘total anxiety and depression’ scores) to facilitate balance to either the FRIENDS or ‘usual provision’ arm of the trial. Usual provision acted as a ‘no‐treatment waitlist’ condition, with the provision that schools within this arm of the trial were not to implement FRIENDS during the period of the trial. Randomization was completed by an independent company specializing in trial allocation.

The initial aim of the study was to recruit 110 classes from 77 schools (an estimate based on the number of single‐entry schools in the implementation area). All calculations assumed: *N* = 28 per cluster (Department for Education, 2015), ICC (class level) = .17, Power = .8, Alpha =.05, proportion of single form entry = 51%. Recruitment exceeded targets, resulting in a total of 128 classes (81 schools), resulting in a minimum detectable effect of .239. Nevertheless, 6 classes (2 schools) did not meet the inclusion criteria for randomization (failure to return minimum level of baseline data), resulting in a total of 122 classes (79 schools). Post‐hoc power calculations, including pre‐test data, give a 2‐tailed minimum detectable effect size (MDES) of .151.

Class was selected as the unit of randomization for several reasons: (i) FRIENDS is typically delivered at the class level, (ii) the implementation by an external delivery agent and internalized context of the content (e.g., controlled breathing in response to internalized cognitions) minimized risks of contamination to comparison classes, (iii) allocation by class allowed most schools to receive the intervention, resulting in favourable pupil: school ratios for purposes of statistical power and continued participation by schools.

The composition of trial schools (located in the South‐East of England) mirrored national averages in terms of size, attendance and average attainment, and the balance on key observables between trial arms was considered to be good as ES differences were observed to be negligible (Table 1). Usual practice schools were monitored for the effects of compensation rivalry through the use of self‐report questionnaires. No significant change in activity was reported (Wigelsworth, 2018).

**TABLE 1:** Characteristics of the randomized classes and pupils (Wigelsworth, 2018).

Class characteristics	National average^b^	Intervention group (*N* = 62) mean (*SD*)	Usual provision (*N* = 60) mean (*SD*)	Balance at randomization (Cohen's *d*, 95% CI)^a^
Sex – percentage of male students within class	50.97	47.01 (14.32)	48.17 (10.72)	−.091 (−.45; .267) (negligible)
Class size	27.1	24.09 (6.78)	25.64 (6.88)	−.227 (−.618; .102) (small)
FSM – proportion of pupils eligible for free school meals within class	.141	.14 (.12)	.16 (.14)	−.129 (−.478; .220) (negligible)
SEND– proportion of pupils with special educational needs and disabilities within class	.135	.14 (.11)	.18 (.14)	−.31 (−.660; .042) (small)

^a^ Thresholds for quantification of effect size defined by Cohen (1992, p. 157). |*d*| < .2 ‘negligible’, |*d*| < .5 ‘small’, |*d*| < .8 ‘medium’, otherwise ‘large’.

^b^ Values drawn from Department of Education (2017).

Baseline measures were taken in February 2016 prior to randomization. Post‐test was taken in December 2016. Data pertaining to the PSWQ‐C and RCADS‐25 were collected simultaneously in a single, counter‐balanced questionnaire pack, utilizing whole‐class administration and a standardized administration protocol. Packs were administered by the intervention agents without the support of teachers.

### FRIENDS

FRIENDS is a whole‐class intervention consisting of 10 weekly sessions of approximately 60–90 min each, which aim to teach and reinforce skills specific to the prevention or reduction of anxiety and depression symptoms and behaviours. For instance, recognition of physiological symptoms of anxiety (e.g., session 2), understanding the relationship between unhelpful thoughts and feelings and their impact on functional or dysfunctional behaviour (e.g., session 4) and the development and practice of a ‘coping step ladder’, increasing exposure to a potential threat with a corresponding practice in coping strategies (e.g., session 7). Additionally, the programme includes two whole‐class booster sessions to facilitate the generalization of FRIENDS to situations encountered in everyday life and incorporates a family‐skills element, including two psychoeducational sessions for parents, approximately 2 hr length each. Parental sessions were not implemented in the evaluation, but all other aspects remained unchanged (see supplementary materials). In the specific context of this study, FRIENDS was delivered exclusively as a universal intervention in year 5 classrooms (ages 9–10). Delivery was by a team of external delivery agents. Intervention agents were all female, aged between 27 and 55 years old (x̄ age 40 years). Two implementers reported that their highest qualification was an Undergraduate Degree or professional equivalent (e.g., BEd) while six implementers had a Postgraduate Certificate (e.g., PGCE). The remaining two implementers reported as their ‘other’ highest qualification (one of them holding a Diploma, while the other did not specify). All implementers had previous professional educational experience in schools (3 out of 10 had between 2 and 5 years of experience, while the remaining seven had 5+ years of experience). Past roles, described as either teaching or pastoral or both, referred to examples such as: teaching PSHE and supporting children who had been excluded and/or school refusers, teaching assistant(s) (behavioural TA/HLTA), early intervention project officer, targeted mental/emotional health project officer, pastoral educational support, supporting self‐esteem in small/large groups, other peer mentoring and anti‐bullying strategies, secondary school teacher and lead in numeracy. Each implementer participated in a one‐day training workshop from a company licensed to deliver FRIENDS training and was otherwise unknown to the class. Classroom teachers were not present during delivery.

FRIENDS is a highly prescriptive, manualized intervention, designed to optimize fidelity of implementation. However, given variable session lengths (in contrast to fixed school timetables) and the context of implementing any ‘real world’ intervention in complex social environments, adaptation is inevitable. Given that there are no agreed thresholds for implementation fidelity (implementation of the intervention was recorded through direct observations of the class by trained researchers), once per implementer (10 observations, carried out in 10 separate classes) and data was then modelled against pupil outcomes. Implementation fidelity was not seen to be related to pupil outcome (Wigelsworth, 2018).

### Participants

A total of 3027 pupils from 122 year 5 classes (81 schools) in England participated in the study. Inclusion criteria required schools to be state‐funded primary schools (e.g., community, foundation or academy) with no previous engagement with FRIENDS. A variety of ethical procedures were implemented: (a) participating schools signed a Memorandum of Agreement (MoA), indicating their willingness to participate, (b) participating schools distributed consent forms to their parents and carers of all eligible pupils (those in year 5 during 2015/2016), via multiple communication channels, including letters, texting services, email and school websites, which contained an ‘opt‐out’ section, and (c) pupil assent was attained. Categories of risk status were created using baseline Revised Children's Anxiety and Depression Scale (RCADS) scores (below).

### Measures

#### Revised children's anxiety and depression scale

The RCADS (Chorpita et al., 2000) is one of the most commonly used measures in adolescent anxiety and depression research (Stallwood et al., 2021), available in both parent‐ and child‐report, and in different item‐number formats, depending on the number of subdomains of clinical concern required. A systematic review and meta‐analysis of over 140 studies using the RCADS shows strong support for its validity and reliability for mental health screening, across a variety of contexts (Piqueras et al., 2017), with additional data supporting its use with British child and adolescent samples (Baron et al., 2021; Challen et al., 2014; Donnelly et al., 2019; Stallard et al., 2014).

The current study utilized the 25‐item version (Muris et al., 2002) which assesses symptoms of anxiety and depression alongside a combined total, which was used for this study.

An example item is: ‘I feel sad or empty’, with responses: ‘never’ = 0, ‘sometimes’ = 1, ‘often’ = 2, and ‘always’ = 3. Cronbach's alpha was .9 for pre‐test and .91 for post‐test. The RCADS has good internal consistency, test–retest stability and convergent and divergent validity (Muris et al., 2002; Sandín et al., 2010). Consistent with the original author's recommendations, individual scores were used to create categories of risk, specifically: normal (*T*‐score below 65); borderline (*T*‐score between 65 and 69); or clinical (*T*‐score 70 or above), for all pupils at baseline and post‐test. Clinical threshold scores were established using the anxiety disorders interview schedule for DSM‐4 (Chorpita et al., 2000).

#### Penn state worry questionnaire for children

The Penn State Worry Questionnaire for Children (PSWQ‐C; Meyer et al., 1990) is one of the most frequently used instruments to assess worry in children (Păsărelu et al., 2017). Comprised of 14 self‐report items, the PSWQ‐C shows strong cultural invariance (Ediati & Utari, 2019; Liu & Zhong, 2020) and has been previously used with English child and adolescent samples (Stallard et al., 2015).

Responses are scored on a 4‐point Likert scale. An example item is ‘My worries really bother me’, with responses: ‘never’ = 0, ‘sometimes’ = 1, ‘often’ = 2, and ‘always’ = 3. Additionally, three items were reversed‐coded. Cronbach's alpha was .88 for baseline and .9 for post‐test. Worry items are suitable for the general population, but act as an indication of potential difficulty as heightened worry is seen as a precursor to the development of anxiety and depression disorders (Rabner et al., 2017).

Worry is a cognitive process that is closely linked to the normal emotion of anxiety (Hirsch & Matthews, 2012), indeed measures of anxiety often include mention of worry (e.g., the Revised Children's Manifest Anxiety Scale (Reynolds & Richmond, 1978) include items such as ‘I worry about what will happen’). Worry is also common to all anxiety disorders (Fresco et al., 2003), which shares a comorbidity with depression disorders in the majority of cases (Hirschfeld, 2001). However, unlike anxiety and depression, worry is not typically measured with respect to clinical thresholds, meaning it is better utilized as a scale response across a sample in assessing (in this context) whether there are any observable intervention effects, with clinical thresholds for anxiety and depression (as measured by the RCADS) used to identify risk status.

## RESULTS

### Descriptives

Missingness was 5.5% and 14.7% for demographic variables and baseline worry scores, respectively. Elevated missingness was observed for post‐test worry scores (23.4%). Where appropriate, missing data were treated using Robust Maximum Likelihood (MLR) with full information. MLR is a commonly used estimation method that calculates a likelihood function for each individual based on the variables that are present so that all the available data are used. The method has been shown to work well across a range of applications, indicating its suitability in addressing concerns as to potential loss of power or misestimation when presented with missing data (Little & Hyonggin, 2003).

Equivalence at baseline was established, with Cohen's *d* indicating <.1 difference in mean baseline outcome measures between trial arms (Table 2). Table 3 shows the frequency distribution and mean PSWQ scores by category of risk status.

**TABLE 2:** Frequencies and PSWQ means by derived risk status.

Measure	FRIENDS x̄ (*SD*)	Comparison x̄ (*SD*)	Balance at randomization (Cohen's *d*)
Baseline RCADS	21.57 (12.21)	20.96 (12.03)	.05 (−.024; .123)
Baseline PSWQ	16.95 (8.46)	16.82 (8.35)	.015 (−.059; .123)

**TABLE 3:** Mean equivalence at baseline.

Risk group (Baseline)	FRIENDS		Comparison	
	Pre‐test (*n* = 1324)	Post‐test (*n* = 1269)	Pre‐test (*n* = 1373)	Post‐test (*n* = 1158)
Non‐clinical	*n* = 1248	*n* = 1151	*n* = 1299	*n* = 1127
x̄ (*SD*)	16.26 (7.98)	15.60 (8.32)	16.24 (7.87)	15.55 (8.84)
Borderline	*n* = 37	*n* = 41	*n* = 25	*n* = 42
x̄ (*SD*)	26.54 (7.18)	24.65 (7.86)	28.94 (6.68)	24.07 (9.51)
Clinical threshold	*n* = 39	*n* = 77	*n* = 39	*n* = 89
x̄ (*SD*)	31.52 (7.19)	22.93 (8.48)	32.22 (6.36)	29.92 (6.29)

### Change in risk status

#### Reduced risk category (i.e., ‘treatment effects’)

Pearson's chi‐square did not indicate a relationship between reducing category of risk and allocation to condition (*χ* ^2^ (1) = 1.667; *p* =.797). To examine change in risk status, pupils were allocated binary codes based on whether scores indicated a move of category risk status between pre‐ and post‐test (e.g., from clinical or borderline to normal). There was no significant difference in the odds of reducing category risk status between FRIENDS and comparison groups (OR = 1.412, 95% CI [.722; 1.807], *n* = 2391). For the highest risk group only (i.e., those in the clinical range at pre‐test), there was no significant difference in the odds of reducing category status (OR = 2.895, 95% CI [1.052; 7.969]).

#### Increased risk category (i.e., ‘preventative effects’)

Pearson's chi‐square did not indicate a relationship between an increasing category of risk and allocation to condition (*χ* ^2^ (1) = .44; *p* =.507). As with examining reduced risk category, binary codes indicating move of category risk status were examined. There was no significant difference in the odds of increasing category risk status between FRIENDS and comparison groups from normal to borderline (OR = .945, 95% CI [.573; 1557]) (*n* = 2963). For borderline to clinical there was no significant difference in the odds of increasing category status (OR = 1.564, 95% CI [.55; 4.405]).

### Impact on worry with risk groups

To examine the impact within each risk group, a multigroup two‐level regression model was fitted in Mplus 8.6 with level 1 representing the pupils (*N* = 3028) and level 2 the classrooms (*N* = 122), with an average cluster size of 19.48 and with an intracluster correlation coefficient of .027 for worry scores at post‐test. Three groups were defined following the RCADS total score representing normal (*n* = 2547, 84.1%), borderline (*n* = 72, 2.4%) and clinical (*n* = 78, 2.6%). Due to 330 missing cases in this grouping variable, the total sample used in the current analysis was 2697. Treatment allocation was added as a level 2 covariate. Baseline worry scores, sex, English as an additional language and free school meal eligibility were added as level 1 covariates. Intervention effects were estimated as *b*/*σ*_e_, consistent with Cohen's *d* thresholds, where *b* represents the treatment beta coefficient and *σ*_e_ corresponds to the standard deviation of worry at post‐test. Results are shown in Table 4.

**TABLE 4:** Two‐level regression of treatment effects on worry scores.

	Groups β (*SE*)		
	Normal	Borderline	Clinical
Level 1: pupils			
Sex (if female)	.10 (.02)***	.32 (.09)***	.34 (.08)***
FSM (if yes)	.02 (.02)	−.10 (.10)	−.17 (.06)**
EAL (if yes)	−.03 (.02)*	−.33 (.11)**	−.05 (.11)
Worry baseline	.49 (.02)***	.35 (.10)***	.36 (.10)***
Level 2: classroom			
Treatment (if FRIENDS)	.03 (.33) *d* < .01	1.03 (.98) *d* = .18	−1.83 (.14)*** *d* = .67

Abbreviations: EAL, english as an additional language; FSM, free school meals.

* *p* < .05.

** *p* < .01.

*** *p* < .001.

After controlling for social demographics (i.e., sex, FSM, EAL) and baseline scores, results showed a treatment effect for the clinical group only (*d* = .67). The borderline group showed a small but non‐significant effect (*d* = .18), and no impact was seen for pupils in the normal group (*d* < .01).

## DISCUSSION

The results of the study found no evidence that FRIENDS was effective in impacting thresholds for anxiety or depressive symptomology either as a treatment or preventative approach. Odds ratios did not detect any change in risk status (as measured by the RCADS) over the course of the study as a result of trial allocation. However, there was evidence for reduced worry symptoms specifically for those within the clinical category for anxiety and depression and, to a lesser extent, those in the borderline category. These findings offer further nuance in interpreting the impact of FRIENDS and advance understanding of optimal treatment and prevention strategies with respect to universal provision, especially with respect to delivery by educational professionals.

Previous literature has called for further examination of FRIENDS for highly anxious children (Stallard et al., 2014), and although the sample size remains small, evidence from the current study was sufficient to provide an indication of increased impact for those identified in the clinical range of anxiety and depression. Results are broadly consistent with international literature that notes smaller effects for lower risk categories; however, this study distinguishes between ‘normal’ and ‘borderline’ categories in contrast to other studies that utilize dichotomous risk groups (e.g., high vs. low risk) (Maggin & Johnson, 2014). We feel that this is an important distinction, as this helps establish the efficacy of FRIENDS with respect to treatment versus prevention modalities. Although odds ratios did not indicate evidence of preventative impact, this was limited by the length of the overall study (11 months) as we would expect evidence of prevention later in the life course for those students in the normal category, as they do not yet have a need to draw upon assets delivered through the FRIENDS programme. Therefore, the reported effect size for those in the borderline category is more closely representative of a treatment effect, in which early symptomology is reduced. Interestingly, the effect size (*d* = .18) for the borderline group is slightly lower than in comparison to previous data (Stallard et al., 2014) (which reports *g* = .23 for children in subclinical categories), especially considering that in the current study, distinguishing the normal group with baseline scores should otherwise elevate the reported effect (by only including those with symptomology).

In consideration of these findings, one possible explanation is that of the background of the implementer. In the current study delivery was conducted by external delivery agents working for a private education company all of whom were from educational backgrounds (e.g., ex‐teachers). Earlier trials comparing health‐led practitioners with education‐based implementers failed to find an impact of FRIENDS when led by educational practitioners (Skryabina et al., 2016) and there remains a debate as to the efficacy of teachers in delivering FRIENDS (Briesch et al., 2010). Results from the current study offer a more optimistic interpretation of education‐led FRIENDS and are consistent with a more general body of literature that suggests that although education‐led implementers are typically associated with smaller effect sizes in comparison to health‐led implementers, effect sizes for psychosocial interventions in addressing worry are around *d* = .13 when delivered by teachers (Franklin et al., 2017). Relatedly, the implementation and process evaluation findings from the original study have important implications for our current analysis of differential impact. The original study reported that quality of delivery was associated with a statistically significant reduction in pupils’ self‐rated worry (ES = −.469, CI = −.523 to −.415), while level of engagement paradoxically showed a smaller but significant effect in the opposite direction (ES = .237, CI = .185 to .289). Results indicate that implementation quality may be a particularly critical factor in the effectiveness of FRIENDS for children with elevated symptoms. The significant treatment effect we observed for the clinical risk group (*d* = .67) may be partly explained by implementation quality factors, as children with higher levels of anxiety and depression may be more responsive to high‐quality delivery of intervention components. The counterintuitive finding regarding engagement (higher engagement associated with slightly increased worry) aligns with our observation that FRIENDS did not demonstrate significant preventative effects. It is possible that increased engagement techniques used by delivery agents may have heightened awareness of anxiety symptoms among students without providing sufficient skills mastery for those not already experiencing difficulties, offering a potential explanation for minimal impact for students in the normal range. Implementation findings reinforce the idea that when delivered by educational professionals, FRIENDS appears more effective as a targeted intervention for those already experiencing clinical symptoms rather than as a universal preventative approach.

This study therefore suggests that, for the specific case of FRIENDS, this may be effectively delivered by teachers to the benefit of some pupils, most notably those reporting in the clinical range for anxiety and depression and, to a lesser extent, those at the borderline level. The current study did not identify an immediate benefit with respect to health‐related outcomes or those in the normal category, although we firmly acknowledge that the trial design did not examine long‐term prevention effects in this respect. In considering generalizability, the study did utilize a nationally representative sample indicating findings may extend across the ‘typical’ classroom context of English primary schools. However, as FRIENDS is considered an international programme, with evidence of its effectiveness drawn from over a dozen different countries (Fisak et al., 2023), and recognition for its global impact (World Health Organisation, 2004). Therefore, findings from the current study may be applicable internationally, indicating that FRIENDS may be helpful for individuals reporting in the clinical range for anxiety and depression and, to a lesser extent, those at the borderline level.

### Limitations and future directions

When interpreting findings, we must exercise appropriate caution, as recommended by Assmann et al. (2000). The differential effects observed across risk categories represent exploratory findings rather than definitive evidence of targeted efficacy. While our analyses detected a meaningful effect size in the clinical risk group (*d* = .67), the relatively small sample size in this subgroup warrants conservative interpretation. The absence of a formal interaction test further limits the strength of conclusions about differential impacts.

Regarding study design, the use of class as the unit of randomization (rather than school) meant that the possibility of contamination between trial arms could not be completely ruled out. The likelihood of contamination was considered low given that the delivery of FRIENDS is by an external implementer and therefore teachers were not present during the delivery of the programme, preventing the adoption and transmission of skills or behaviours by the class teacher. Peer‐to‐peer contamination remains a possibility, though the nature of the intervention did not include the overt demonstration of behaviours (e.g., controlled breathing is a personal rather than social behaviour) or encourage or promote the use of behaviours for use with others (e.g., no social skills training), reducing opportunities for overt demonstration of skills and replication by the comparison group (e.g., on the playground). The risk of contamination was offset by the substantial advantages of reduced trial and implementation costs by having FRIENDS and usual practice classes on the same site with corresponding security in improved retention and minimization of potential compensation rivalry (as double, triple and four‐form entry schools were in receipt of FRIENDS for at least some classes).

Regarding measures, the study did not directly measure the skills that FRIENDS is designed to teach and instead captured evidence of symptom change/reduction. Evidence of symptom change is theorized to be consistent with the underlying mechanism of change of the intervention (i.e., deploying behavioural and cognitive responses to intra‐personal and/or environmental stimuli), however future research would benefit from directly examining the acquisition and maintenance of the skills taught themselves.

The current findings indicate that whereas FRIENDS did not appear to facilitate a change in risk status, there is evidence of treatment effects, most notably for those in the clinical range of difficulties. This suggests that, in contrast to some earlier literature, FRIENDS may be suitable for delivery by educational professionals. However, in the classroom context, there is also the consideration as to the cost–benefit of preventative effects of FRIENDS and the suitability of educators as delivery professionals should be weighed against other factors such as availability of other support and treatment services, relative cost and sustainability.

## AUTHOR CONTRIBUTIONS

**Garry Squires:** Conceptualization; investigation; funding acquisition; writing – review and editing. **Karolina Byc:** Methodology; writing – original draft. **Margarita Panayiotou:** Formal analysis; data curation; writing – review and editing. **Michael Wigelsworth:** Conceptualization; investigation; funding acquisition; writing – original draft; project administration.

## FUNDING INFORMATION

This research was supported in part by funding from the Education Endowment Foundation.

## CONFLICT OF INTEREST STATEMENT

On behalf of all authors, the corresponding author states that there is no conflict of interest.

## Data Availability

The data that support the findings of this study are available from the corresponding author upon reasonable request.

## APPENDIX A Description of the FRIENDS programme

In order to provide a comprehensive and transparent description of the FRIENDS programme, we utilize an adapted version of the Template for Intervention Description and Replication (TIDieR) (Hoffmann et al., 2014).

### A.1. Name of intervention

‘FRIENDS for Life’ (hereafter referred to as ‘FRIENDS’).

‘FRIENDS for Life’ is one of the three versions of the FRIENDS curriculum (in contrast to FUN FRIENDS; ages 4–7, and My FRIENDS youth; ages 12–16), and is designed specifically for ages 8–11 years.

FRIENDS is an acronym, broadly reflecting the programme content (**F**eelings; Remember to relax; **I**nner helpful thoughts; **E**xplore solutions and coping plans; **N**ow reward yourself; **D**o it every day; **S**tay strong inside).

### A.2. Why (rationale/theory)

Throughout various updates in content, the theoretical model underpinning FRIENDS can be consistently described as cognitive behaviour therapy (CBT). Based on the work of Aaron Beck (1964), CBT links thoughts with feelings and behaviour.

By acquiring, reinforcing, and maintaining specific skills and behaviours, individuals can learn to recognize when their thoughts are unhelpful, and this can lead to more helpful thinking, moderation of feelings or changes in behaviour. The programme therefore teaches children skills in the following domains: cognitive (identify and challenge anxiety‐increasing cognitions), emotional (identify and manage anxiety) and behavioural (problem‐solving and face‐feared situations). The theoretical model for the prevention and early intervention for anxiety in specific relation to FRIENDS is shown in appendix A. CBT has been described as the ‘gold standard’ in the treatment of anxiety disorders (Otte, 2011, p. 413); however, this recommendation has been largely based on clinical treatment in adults, with far less known about its utility as a preventative approach with children.

### A.3. Who (recipients)

FRIENDS is a universal intervention, and the iteration of FRIENDS delivered as part of this trial was delivered in whole‐class settings. It can be adapted for use as a selective and targeted intervention as either an alternative or as an augment to universal delivery. In the specific context of this trial, FRIENDS was delivered exclusively as a universal intervention to year 5 classrooms.

### A.4. What (materials)

Individuals responsible for implementing FRIENDS are provided a single copy of the group leader's manual to accompany the pupil workbooks (one per pupil). The group leader's manual provides a highly prescriptive syllabus, including session goals, verbatim scripts and activities lasting between 5 and 10 min each. The pupil workbook provides template activities aligning to the exercises in the group leader's manual. For example, pupils have to look at a series of pictures and using different coloured pencils to write ‘feeling’ words; then think about and write other feelings. An overview of session content can be seen in Table A1.

**TABLE A1:** Overview of FRIENDS sessions.

Session	Examples of behavioural change
1. Understanding feelings in ourselves and others	Increase in ability to recognize and name own emotions
	Increase in ability to recognize emotions in others
	Understand that different feelings depend on the situation AND what we think
	How we feel affects our behaviour
2. Introduction to feelings	Increased ability to identify, name and express feelings in self and others
	Guided discovery and relaxation increase awareness of bodily sensations and activation of the parasympathetic nervous system
3. Introduction to body clues and relaxation	Describe bodily changes associated with sympathetic and parasympathetic nervous system activation
	Practice parasympathetic nervous system activation through controlled breathing; using progressive muscle relation (PMR); continuing with guided discovery
4. Helpful and unhelpful thinking	Differentiating between thoughts and feelings
	Understanding that thoughts and feelings are linked
	Identifying ways that we can think differently in a given situation and how this impacts our feelings
	Understanding that some thoughts are helpful or adaptive and others are unhelpful or maladaptive and these then lead to behaviours which are functional or dysfunctional
	Start to consider how to use evidence to challenge unhelpful thoughts and support helpful thoughts
	Practice the use of challenger thoughts, for example, ‘stop – think’
5. Changing unhelpful thoughts into helpful thoughts	Adaptive and maladaptive thoughts are framed as helpful thoughts and unhelpful thoughts. Class activities are aimed at improving children's ability to describe thoughts as helpful or unhelpful in a given situation
	Further practice on the use of challenger thoughts (stop – think) Further use of evidence to challenge unhelpful thoughts and support helpful thoughts
	Continued practice of guided discovery and PMR
6. Introduction to coping step plans	Develop a coping step plan which is based on the ‘Fear Ladder’ in CBT. The structure involves the child in identifying how to increase exposure to a potential threat in a stepped way such that they are increasingly able to cope
	Continued practice of PMR
7. Learning from our role models and building support teams	Sharing of coping plans leads to social learning of coping strategies
	Children identify who could be part of their social support network
	The coping plan is developed further to include social support.
	Implementation of the revised stepped coping plan with social support
8. Using a problem‐solving plan	The children learn 6 steps to solving problems such as focusing on how the problem is defined in terms of ‘what I think’, ‘what else I could think’, ‘how do I know which thought is right’
	This leads to behavioural experiments in which the children can practice the 6‐step problem‐solving technique
	Further practice of PMR and guided discovery
9. Using the FRIENDS skills to help ourselves and others	The aim is to practice the skills so far
	Emphasis on the problem‐solving plan and coping step plan
	Thinking about how to use social support systems
	Practising PMR and guided discovery
	Implementing coping step plans and problem‐solving techniques
	New behaviours include: Noticing success Task analysis to note partial success Being part of a social support system for others Identifying goals and rewards Directing attention to what works well (and away from perceived threats) Understanding ruminating thoughts and negative automatic thoughts and how these can be replaced with more helpful thoughts Thinking about how social support networks can help to develop the maintenance of new skills
10. Review and party	Maintenance and relapse prevention
	Social support network affirmations
Booster 1 3–4 weeks after the main programme	Maintenance and relapse prevention
Booster 2 3–4 weeks after Booster 1	Maintenance and relapse prevention

### A.5. What (procedures)

FRIENDS is a taught syllabus. FRIENDS consists of 10 weekly sessions, which aim to promote various protective factors, such as recognizing physiological symptoms (e.g., session 2), emotional self‐management (e.g., session 4) and supporting peer relationships (e.g., session 8). Indeed, the name of the programme is designed to help pupils recall and utilize the various skills taught in the sessions (as above). Each session is designed to last 60–90 min, but the manual recommends a delivery model of two 30–35 min periods instead. Additionally, there are two 1‐h whole‐class booster sessions reinforcing the application of FRIENDS to real‐life situations that can be held approximately 1–3 months after completing the programme, and 2 information sessions for parents of approximately 2 h length each. Booster sessions were completed, but parental sessions were not. There have been revisions across the lifespan of the intervention in response to feedback received from recipients and stakeholders. Later editions (used from 2010 onwards) provide more activities to increase the flexibility of programme delivery. New content has been included to cover aspects of resilience (e.g., attention training, diet and exercise) and an increased focus on addressing externalizing difficulties (Barrett, Cooper & Guajardo, 2014).

As the implementation of FRIENDS is encapsulated within an existing delivery team (see 6. Who), there are minor adaptations and additions specific to the context. First, there is a pre‐implementation phase in which the delivery agent (commonly known as ‘coach’, or in this specific context, ‘project officer’) forms a relationship with a member of the school. Second, parental lessons are not implemented in this specific trial. Although part of the original intervention, parental sessions have not been identified as a core component of the intervention and have been omitted. All other aspects of the intervention remained unchanged.

### A.6. Who (provided)

For this trial, FRIENDS was implemented by an external delivery team. One implementer was responsible for delivering FRIENDS in up to 10 different schools.

### A.7. How

FRIENDS is explicitly timetabled during the normal school day.

### A.8. Where

FRIENDS was implemented in school, within classrooms.

### A.9. When and how much

As noted above, FRIENDS is delivered through 10 weekly sessions. The length of each weekly session varies (e.g., between 60 and 90 min) and therefore, there is not a precise mapping of week = activity, as implementers are required to deliver the intervention within the constraints of the school timetable. This means that the dosage of specific activities listed in the instructor's manual may vary (being truncated or omitted as time allows) and/or sessions carried over to later weeks.

### A.10. Tailoring

FRIENDS is a highly prescriptive, manualized intervention, designed to optimize the fidelity of implementation. However, given variable session lengths (in contrast to fixed school timetables) and the context of implementing any ‘real world’ intervention in complex social environments, adaptation is inevitable. This typically takes the form of truncation (or omission), substitution or expansion of activities by implementers.

### A.11. How well (planned)

Implementers receive 1 day of training. This training is only available to staff who are already working with children and young people in a professional capacity (e.g., teachers, pastoral support workers, youth workers). In the context of the current trial, training was provided by ‘Interactive Connections’, a licensed training company (now retired). The company providing the implementers was licensed to both train and provide licensed facilitators.
